# Insights into Mobile Genetic Elements of the Biocide-Degrading Bacterium *Pseudomonas nitroreducens* HBP-1

**DOI:** 10.3390/genes11080930

**Published:** 2020-08-12

**Authors:** Nicolas Carraro, Vladimir Sentchilo, Lenka Polák, Claire Bertelli, Jan Roelof van der Meer

**Affiliations:** 1 Department of Fundamental Microbiology, University of Lausanne, Biophore, Quartier UNIL-Sorge, 1015 Lausanne, Switzerland; vladimir.sentchilo@unil.ch (V.S.); lenka.polak@chuv.ch (L.P.); janroelof.vandermeer@unil.ch (J.R.v.d.M.); 2 Institute for Microbiology, Lausanne University Hospital and University of Lausanne, Bugnon 48, 1011 Lausanne, Switzerland; claire.bertelli@chuv.ch

**Keywords:** *Pseudomonas azelaica*, integrative and conjugative elements, ICE*clc*, aromatic compounds, heavy metal, adaptation, evolution

## Abstract

The sewage sludge isolate *Pseudomonas nitroreducens* HBP-1 was the first bacterium known to completely degrade the fungicide 2-hydroxybiphenyl. PacBio and Illumina whole-genome sequencing revealed three circular DNA replicons: a chromosome and two plasmids. Plasmids were shown to code for putative adaptive functions such as heavy metal resistance, but with unclarified ability for self-transfer. About one-tenth of strain HBP-1′s chromosomal genes are likely of recent horizontal influx, being part of genomic islands, prophages and integrative and conjugative elements (ICEs). *P. nitroreducens* carries two large ICEs with different functional specialization, but with homologous core structures to the well-known ICE*clc* of *Pseudomonas knackmussii* B13. The variable regions of ICE*Pni*1 (96 kb) code for, among others, heavy metal resistances and formaldehyde detoxification, whereas those of ICE*Pni*2 (171 kb) encodes complete *meta*-cleavage pathways for catabolism of 2-hydroxybiphenyl and salicylate, a protocatechuate pathway and peripheral enzymes for 4-hydroxybenzoate, ferulate, vanillin and vanillate transformation. Both ICEs transferred at frequencies of 10^−6^–10^−8^ per *P. nitroreducens* HBP-1 donor into *Pseudomonas putida*, where they integrated site specifically into *tRNA^Gly^*-gene targets, as expected. Our study highlights the underlying determinants and mechanisms driving dissemination of adaptive properties allowing bacterial strains to cope with polluted environments.

## 1. Introduction

The recent industrial revolution has driven the intensive exploitation of natural resources and heavy use of chemicals, leading to massive environmental pollution. Besides its potential harmful effects on human health, pollution also strongly impacts the functionality of ecosystems. Even at the microbial level, polluting substances pose a strong selection, leading to the disappearance of sensitive strains, and the enrichment of adapted and resistant strains [1]. In some instances of organic pollution, strains have evolved to metabolize and transform the compounds under scrutiny [1]. Pollutant-degrading bacteria have attracted interest, because they may be applied to directly or indirectly remediate pollution and restore ecosystem health [1,2]. The inoculation of specific strains, such as *Pseudomonas* sp. JS150, *Pseudomonas knackmussii* B13, or *Pseudomonas veronii* 1YdBTEX2, has been shown to significantly enhance biodegradation of pollutants such as phenol, chlorobenzoate, benzene, toluene or xylene [3,4,5].

*Pseudomonas nitroreducens* HBP-1 (DSM 8897, previously named *Pseudomonas azelaica* HBP-1), was isolated in 1988 from sewage sludge in California [6], and is one of the rare bacteria able to completely degrade and use the biocide 2-hydroxybiphenyl (2HBP) as the sole carbon and energy source [6]. The metabolic pathway for 2HBP conversion in *P. nitroreducens* HBP-1 has been biochemically and genetically characterized in detail [6,7,8,9,10,11]. The enzymes responsible for the first steps of 2HBP metabolism are encoded by the *hbpCAD* locus. A broad-spectrum monooxygenase (HbpA) catalyzes the hydroxylation of 2HBP and a wide range of other 2-substituted phenols to their corresponding catechols [10]. Subsequent action of a dioxygenase (HbpC) leads to the cleavage of the hydroxylated ring, which is then converted to benzoate and 2-hydroxy-2,4-pentadienoic acid by means of the HbpD hydrolase. Both compounds are further metabolized via *meta*-cleavage degradation pathways. The expression of the *hbpCAD* genes is regulated at the transcriptional level by the sigma-54-dependent activator HbpR in the presence of 2HBP (or analogous molecules) [12,13,14].

The genomes of *P. nitroreducens* HBP-1 as well as its close relative *P. azelaica* strain Aramco J were recently released as drafts, consisting of 212 and 91 contigs, respectively [15,16]. Unfortunately, although the draft genomes provided insights into the gene content and functional capacities of *P. nitroreducens*, their incompleteness hampered comparative genomics analyses and evaluations of the potential role of mobile genetic elements in their evolution. The main goal of this study was, therefore, to produce a complete gapless genome of *P. nitroreducens* HBP-1 and to study the nature and functionalities of its potential mobile elements. We used a combination of Illumina short-read sequencing as well as PacBio long-read sequencing to assemble the HBP-1 genome. Regions of genome plasticity in HBP-1 were further analyzed for characteristics of genomic islands, putative prophages, and integrative and conjugative elements (ICEs). Mating assays between *P. nitroreducens* HBP-1 as donor and *P. putida* UWC1 as recipient were conducted to examine self-transfer of deduced ICEs. We found that *P. nitroreducens* contains two active ICEs that can transfer at low rates. The ICEs carry extensive sets of genes involved in aromatic compound metabolism and resistance to heavy metals, likely determining the ability of strain HBP-1 to cope with numerous toxic compounds. The capacity to spread adaptive functions via the transfer of ICEs could favor adaptation of further bacterial members in natural communities to withstand pollutant stress [17].

## 2. Materials and Methods

### 2.1. Culture Conditions

*P. nitroreducens* HBP-1 (formerly *P. azelaica* HBP-1) [6] and *P. putida* UWC1 miniTn7::*P_tac_-mcherry* (UWCGC) (Gm^r^) [18] were routinely grown in lysogeny broth (LB Miller, Difco) at 30 °C and 180 rpm in an orbital shaker/incubator. The strains were preserved at −80 °C in LB containing 15% (vol/vol) glycerol. As a defined medium, 21C minimal medium [19] was used, which was supplemented with one of the following carbon sources (final concentrations): sodium succinate (20 mM), 2-HBP (2.5 mM, dosed 1:100 from a stock in dimethylsulfoxide), salicylate (2.5 mM), benzoate (2.5 mM), 4-hydroxybenzoate (2.5 mM), vanillate (2.5 mM), phthalate (2.5 mM), phenol (2.5 mM), or catechol (2.5 mM). Whenever necessary for the selection of genetic constructs, gentamicin (10 or 20 µg mL^−1^), rifampicin (25 µg mL^−1^), mercuric chloride (4 µM) or sodium arsenite (5 mM) were amended to the growth media.

### 2.2. DNA Isolation, Sequencing and Assembly

The *P. nitroreducens* HBP-1 genome was sequenced with both long-read (PacBio, Menlo Park, CA, USA) and short-read (Illumina paired-end) technology. For PacBio sequencing, a single clone grown from a glycerol stock of *P. nitroreducens* HBP-1 on a minimal medium–succinate plate, was inoculated into the same liquid medium and was harvested in mid-exponential growth phase (ca. 0.5 optical density units at 600 nm). Four aliquots of ca. 10^9^ cells were pelleted by centrifugation at 14,000× *g* for 5 min, and DNA was extracted in parallel using a PowerSoil DNA extraction kit (MoBio Laboratories, Carlsbad, CA, USA). The resulting DNA aliquots were pooled together, precipitated with ethanol-sodium acetate, washed with 75% ethanol, briefly dried and dissolved in 5 mM Tri-HCl pH 8. DNA quality was analyzed on a 2100 Electrophoresis Bioanalyzer Instrument (Agilent Technologies, Santa Clara, CA, USA), and quantified with an Invitrogen™ Qubit™ 3 Fluorometer (Thermo Fisher Scientific Inc, Waltham, MA, USA). Subsequently, 7.4 μg of DNA were fragmented at 4100 rpm for 1 min with a Covaris g-TUBE device (Covaris Ltd., Brighton, UK) and 5 μg was used for preparing sequencing libraries (SMRTbell template prep kit 1.0, Pacific Biosciences, Menlo Park, CA, USA). DNA sequencing was performed on a PacBio RSII instrument (Pacific Biosciences, Menlo Park, CA, USA) at the Lausanne Genomic Technologies Facility using a single v3 SMRT™ Cell, P6-C4 chemistry and 4-h movies time. A total of 52,338 reads with a mean length of 13,833 nt were de novo assembled and circularized using the Hierarchical Genome Assembly Process (HGAP) version 3.0 [20], yielding three circular replicon assemblies with 83-fold average coverage. The assembly was validated using Illumina 50-nt paired-end sequence reads, obtained on a library prepared and sequenced essentially as described by Miyazaki et al. [4] in a single flow lane using the Illumina Genome Analyzer II sequencing platform, resulting in 8,000,273 reads. Subsequently QC-trimmed reads were mapped to the HGAP assembly using Bowtie [21], the output was converted to SAM/BAM format with SAMtools [22] and the assembly was validated with QualiMap version 2.2.1 [23,24].

In order to quantify and compare mean coverages of the three *P. nitroreducens* HBP-1 replicons, we resequenced another two DNA samples prepared from two succinate grown cultures inoculated either with a single or with five independent *P. nitroreducens* HBP-1 colonies. DNA was extracted from 10^9^ cells by bead beating and magnetic bead purification (CleanNGS beads, LABGENE Scientific SA) following the manufacturer′s protocol. Indexed libraries were constructed by using the Vazyme TruePrep DNA Library Prep kit V2 for Illumina (Vazyme) starting from 1 ng DNA input. Libraries were paired-end sequenced on a Illumina HiSeq2500 with HiSeq Rapid SBS Kit V2 in a rapid 2 × 150 mode.

### 2.3. Annotation

The finished assembly of *P. nitroreducens* strain HBP-1 was annotated by the NCBI Prokaryotic Genome Annotation Pipeline (PGAP) [25,26] and deposited to GenBank (see below for accession numbers). In addition, Rapid Annotation using Subsystems Technology (RAST) [27] and Prokka [28] annotations were explored for maximum details about gene content. The functional gene content of the reported mobile genetic elements was inspected manually and, whenever needed, used to improve the automatic annotations based on analyses by BLAST to GenBank and UniProt Knowledge Base (UniProtKB) with the emphasis given to expert annotations. KEGG and MetaCyc [29], or specific literature were used as reference for metabolic pathways.

### 2.4. In Silico Analyses of Genomic Islands, ICEs and Prophages

IslandViewer [30] and PHASTER [31] were used with default parameters to search for genomic islands and phages, respectively. The data were visualized using ACT Artemis [32], DNA plotter [33], and Adobe Illustrator (Adobe Inc, San Jose, CA, USA, vs. 2020).

### 2.5. Characterization of ICE Attachment Sites

PCR primers were designed to amplify putative *attL* and *attR* recombination sites of ICE*Pni*1 and ICE*Pni*2 in *P. nitroreducens* HBP-1 and amplicons were verified by amplicon sequencing (Table 1). The primer sets used to amplify the specific attachment sites in *P. nitroreducens* and *P. putida* are listed in Table 2. PCRs were performed using 1 × GoTaq^®^ G2 Green Master Mix (Promega), with 100 pM of each primer and 20 ng of purified DNA as a template with the following thermal cycler conditions: (i) 3 min at 94 °C; (ii) 30 cycles of 30 sec at 94 °C, 30 sec at the appropriate annealing temperature, and 1 min per kb at 72 °C; and (iii) 5 min at 72 °C. After agarose gel analyses, the intended PCR products were purified using a NucleoSpin^®^ Gel and PCR Clean-up kit (Macherey-Nagel, Düren, Germany), and Sanger-sequenced at GATC Services (Eurofins Genomics, Luxembourg, Luxembourg). Nucleotide sequences were aligned and compared using DNASTAR Lasergene v.15 package and BLASTn.

### 2.6. ICE Transfer Assays

The ability of the discovered ICEs to self-transfer was examined in conjugation assays between *P. nitroreducens* HBP-1 (donor) and *P. putida* UWC1 miniTn7::*P_tac_-mcherry* (recipient) [18]. For ICE*Pni*1 transfer, the donor was grown in liquid minimal medium with 20 mM succinate supplemented with either 4 mM sodium arsenite or 8 µM mercury chloride. For ICE*Pni*2, the donor was grown in liquid minimal medium with 5 mM 2-HBP. The recipient was grown in succinate medium amended with 20 µg mL^−1^ Gm and 25 µg mL^−1^ Rif. Both cultures were harvested at OD_600_ 0.5–1.0 by centrifugation for 5 min at 5000× *g*, washed once in 1 mL minimal medium, combined at 1:1 donor:recipient ratio, pelleted by centrifugation as above, resuspended and then deposited on a 0.2–µm cellulose acetate filter (Sartorius) placed on 1 mM succinate minimal media agar. Plates with filters were incubated for 48 h at 30 °C. The number of donor and recipient cells per mating ranged between 4.1 × 10^7^ and 7.5 × 10^8^ (per filter). After the incubation, the resulting mating mixes were resuspended in 0.5 mL of minimal medium, serially diluted and plated on selective media as follows. The number of donor cells (*P. nitroreducens* HBP-1) was enumerated on minimal medium agar plates supplemented with 2.5 mM 2HBP. Colonies of the UWCGC recipient (*P. putida* UWC1 miniTn7::*P_tac_-mcherry*) were counted on minimal media agar amended with 20 mM succinate, 20 µg mL^−1^ gentamicin and 25 µg mL^−1^ Rif. *P. putida* ICE*Pni*1-exconjugants were selected on minimal medium agar plates supplemented with 20 mM succinate, 20 µg mL^−1^ gentamicin and 4 µM mercury chloride, whereas, for ICE*Pni*2 exconjugants, the minimal medium was amended with 20 µg mL^−1^ gentamicin and either 5 mM 2HBP or 2.5 mM salicylate. The exconjugant colonies were purified by streaking on the respective selective media and the presence of ICEs was confirmed by PCR as follows. The ICE*Pni*1 marker *merA* was amplified with primer pair pPazICE1_merA_fw and pPazICE1_merA_rev, whereas ICE*Pni*2 marker *hbpA* was amplified with pPazICE2_hbpA_fw and pPazICE2_hbpA_rev (Table 1). In addition, the *P. putida* background of putative exconjugants was confirmed by mCherry fluorescence detected with a Zeiss Axioplan II microscope equipped with a 100× Plan Achromat oil objective lens (Carl Zeiss, Oberkochen, Germany), a SOLA SE light engine (Lumencor, Beaverton, OR, USA), and a SPOT Xplorer 1.4 Mp Cooled CCD Camera (SPOT Imaging Solutions, a division of Diagnostic Instruments, Inc., Sterling Heights, MI, USA).

### 2.7. Database Submission

The complete gapless *P. nitroreducens* HBP-1 genome is available from Genbank under accession numbers CP049140, CP049142, CP049141 for the chromosome, and the two plasmids pPniHBP1_1 and pPniHBP1_2, respectively.

## 3. Results and Discussion

### 3.1. Genome of Pseudomonas Nitroreducens HBP-1

**Figure 1:**
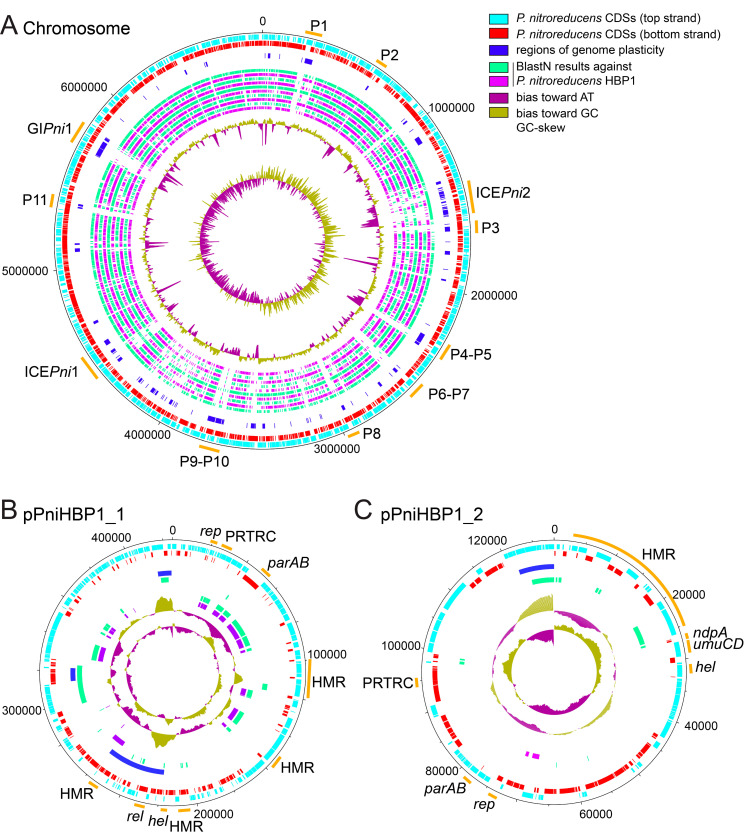
Circular maps of replicons of the *P. nitroreducens* HBP-1 genome. (**A**). Chromosome with predictions of the main genomic island (GI*Pni*1), prophages (P) and ICEs (ICE*Pni*1 and ICE*Pni*2). The outermost circles show the location of predicted ORFs on the top strand (light blue) and bottom strand (red), followed by IslandViewer and PHASTER predictions showing regions of genome plasticity (dark blue). The inner circles represent BlastN comparisons with genomes of other *Pseudomonas* species (alternating green and purple circles), from the outside to the inside: *Pseudomonas* sp. AK6U (Acc. No., NZ_CP025229.1), *Pseudomonas knackmussii* B13 (NZ_HG322950.1), *Pseudomonas putida* KT2440 (NC_002947.4), *Pseudomonas aeruginosa* PAO1 (NC_002516.2), *Pseudomonas citronellolis* SJTE-3 (NZ_CP015878.1), *Pseudomonas oryzihabitans* USDA-ARS-USMARC-56511 (NZ_CP013987.1), *Pseudomonas pseudoalcaligenes* CECT 5344 (NZ_HG916826.1), *Pseudomonas resinovorans* NBRC 106553 (NC_021499.1), *Pseudomonas stutzeri* 28a24 (NZ_CP007441.1), and *Pseudomonas veronii* 1YdBTEX2 (LT599583.1). The two innermost circles depict the GC plot (penultimate circle), and the GC skew (most central circle). Numbers around the black circle indicate the size in base pairs. (**B**). Plasmid pPniHBP1_1 with the indication of heavy metal resistance loci (HMR), active partition system (*parAB* or PRTRC), initiation replication protein (*rep*), relaxase (*rel*), helicase (*hel*). Organization is the same as that described for the map of the chromosome except for the BlastN comparisons, which were performed with (from the outside to the inside) *Pseudomonas putida* KT2440 (NC_002947.4), *Pseudomonas aeruginosa* genomic island PAGI-5 (EF611301.1), *Pseudomonas aeruginosa* strain PA298 plasmid pBM908 (CP040126.1), and *Pseudomonas aeruginosa* strain T2101 plasmid pBT2101 (CP039991.1). (**C**). Plasmid pPniHBP1_2 with the same indications as for pPniHBP1_1, and other features of interest (*umuCD*, *ndpA*). Organization is the same as that described for the map of the chromosome except for the BlastN comparisons, which were performed with (from the outside to the inside). *Pseudomonas* sp. SCB32 chromosome (CP045118.1), *Pseudomonas aeruginosa* strain AR_0356 plasmid (pAR0356, CP027167.1).

A complete genome of *P. nitroreducens* HBP-1 was sequenced using PacBio long read technology, assembled with HGAP and annotated by the NCBI Prokaryotic Genome Annotation Pipeline (PGAP) [25,26]. The final gapless *P. nitroreducens* genome is composed of three replicons: a circular chromosome of 6874,118 bp (CP049140), and two plasmids pPniHBP1_1 (CP049142, 425,042 bp) and pPniHBP1_2 (CP049141, 128,388 bp, Figure 1 and Table S1, sheet GenomeFeatures). Remapping of Illumina sequence reads on the complete assembled genome showed that the three replicons have roughly the same coverage, suggesting a single copy of each replicon per cell (Figure S1). Comparison with a previously published draft genome of HBP-1 [15] notably highlighted the presence of a third replicon (pPniHBP1_2), whereas only two repicons (a chromosome and a megaplasmid) had been proposed previously [15]. Further analysis indicated that the sequences of all three replicons (Figure 1) are found among the 212 contigs of the draft genome but had remained fragmented. Some of the previous contigs overlap the (arbitrary) starts and ends of the two newly assembled replicons, confirming that *P. nitroreducens* plasmids are closed circular DNA molecules (Figure 1A,B). The chromosome of *P. nitroreducens* aligned well with those of a number of other *Pseudomonas* species but presented a variety of regions of genome plasticity that likely correspond to genomic islands (Figure 1A, green and purple circles; discussed further below).

### 3.2. Genomic Insight into Plasmids of *P. nitroreducens*

*P. nitroreducens* HBP-1 carries two plasmids that were named pPniHBP1_1 and pPniHBP1_2 (Figure 1B,C). pPniHBP1_1 is circa 425 kb and codes for a putative RepB replication initiation protein G5B91_33395 [35]. At least two putative partition systems are encoded: a *parA*/*parB* locus (G5B91_33565/G5B91_33570), a gene coding for a PRTRC system ParB protein (G5B91_33435) and two orphan ParB-encoding genes (G5B91_35220 and G5B91_33555, Figure 1B and Table S1 pPniHBP1_1). pPniHBP1_1 also codes for a putative relaxase VirD2 (Figure 1B), but no other conjugative transfer components, suggesting that it could be a mobilizable plasmid relying on the T4SS of another autonomous conjugative element for its transfer [36]. pPniHBP1_1 carries three loci coding for resistance to heavy metals: copper, tellurite, mercury and arsenate/arsenite (Figure 1 and Table S1, sheet pPniHBP1_1).

Plasmid pPniHBP1_2 is 128,388 bp in length and encodes a RepB-type replication initiation protein, a ParA family protein, a ParB/RepB/Spo0J family partition protein and a replication terminus site-binding protein. All share about 50–65% aa identity with homologs from other *Pseudomonas* plasmids. Other plasmid-related functions include six ParB-homologs, a ThiF-Related Cassette (PRTRC), an abortive phage infection protein, a type-I restriction-modification system DNA methylase and a type IV toxin-antitoxin gene pair (AbiEi/AbiEii) (Table S1, sheet pPniHBP1_2). In addition, pPniHBP1_2 carries a 12-gene cluster that is well conserved among other *Pseudomonas* plasmids (notably, the *P. resinovorans* plasmid pCAR1). It seems unlikely that pPniHBP1_2 encodes its own conjugal transfer system since no hallmark type IV secretion system (T4SS) structural genes were identified. We did find genes for the putative conjugal transfer mating pair stabilization proteins TraN, the PilB T4P pilus assembly pathway ATPase-like protein, a helicase and an endonuclease (Table S1 pPniHBP1_2). This might indicate that pPniHBP1_2 is not self-transferable, but would depend on other mobile genetic elements for transfer [36]. The functional gene cargo on pPniHBP1_2 is predicted to code for proteins involved in sensing and defense by the efflux of heavy metal cations such as Co^2+^, Zn^2+^, Cd^2+^, Cu^2+^, Ag^1+^, Hg^2+^, Pb^2+^ (Table S1, sheet pPniHBP1_2) [37,38,39]. The plasmid is also predicted to code for the metabolism of diverse (amino)aromatic compounds (Table S1, sheet pPniHBP1_2).

### 3.3. Genomic Islands in the Genome of *P. nitroreducens*

The analysis of regions of genome plasticity in the *P. nitroreducens* chromosome by Island Viewer [30] and PHASTER [31] indicated several potential genomic islands (GIs) and prophages (Figure 1A). Moreover, the two plasmids might contain GIs (Figure 1B,C). The *P. nitroreducens* genome likely contains eight intact prophages (P1, P2, P6, P7, P8, P9, P10, P11), whereas a further seven regions (P3, P4, P5, P12, P13, P14, and P15) may encode satellite prophages, phage remnants or tailocins [40,41] (Figure 1A and Table 3). None of the phage-related GIs appeared to code for obvious adaptive functions.

Two GIs (Figure 1A) contained homologs to the hallmark gene VirB4 from the T4SS and, therefore, encompassed potentially transferable elements [42]. The product of both VirB4 homologs had a high similarity to the VirB4 protein of the prototypical element ICE*clc* of *P. knackmussii* B13, suggesting they belong to a mating-pair formation type MPF_G_ that is characteristic for ICE T4SS [42]. BlastP comparison with VirB4_ICE*clc*_ revealed 95% of amino acid (aa) identity over 98% of the protein sequence for VirB4_ICE*Pni*1_, and 79% of aa identity over 99% for VirB4_ICE*Pni*2_. The GIs that encompassed the *virB4* homolog genes were thus renamed as putative ICEs ICE*Pni*1 and ICE*Pni*2 (integrative conjugative element of *P. nitroreducens* 1 and 2) for reasons outlined further below. Although ICE*Pni*2 had been detected before and named ICE*bhp* [15], it was renamed here to comply with the suggestions put forward by Burrus and collaborators [43].

The third GI encompassed a 123-kb region between *tRNA^Gln^* and *tRNA^Met^* (G5B91_27170 and 27745) on the *P. nitroreducens* HBP-1 genome (Figure 1A, designated as genomic island *P. nitroreducens* 1, GI*Pni*1). GI*Pni*1 did not contain any genes coding for horizontal transfer, thus preventing a more specific classification (Figure 1A and Table S1, sheet GI*Pni*1). Sequence analysis revealed that GI*Pni*1 bears two hallmark proteins shared by prophages and ICEs: a tyrosine type recombinase/integrase (G5B91_27195) and an AlpA-related transcriptional regulator/excisionase (G5B91_27730). Further resemblance to prophages was limited to three proteins: an inovirus Gp2 family protein, and two DUF3732 and DUF932 domain-containing proteins (Table S1, sheet GI*Pni*1). There was little conservation in gene content between GI*Pni*1 and other putative GIs occupying the same genome plasticity region in other *Pseudomonas* genomes, except for about a dozen genes surrounding the integrase gene and few other hypotheticals scattered throughout the GI (Table S1, sheet GI*Pni*1). The large majority of the functions encoded on GI*Pni*1 are enzymes associated with lipid metabolism. This concerns mainly fatty acid beta-oxidation pathway enzymes, many of which are represented by multiple alleles, for example, (long- and medium-chain) fatty acid-CoA ligases (4 alleles), acyl-CoA dehydrogenases (12), enoyl-CoA hydratases (8), thiolases (2), oxidoreductases (14), (acyl) CoA-transferases (3 alleles, Table S1, sheet GI*Pni*1). Their corresponding genes are organized in operon-like structures and are associated with transcriptional regulators, transporters and electron-transfer components, likely forming complete and functional metabolic systems. In addition, the GI*Pni*1 encodes a dozen different hydrolases and amidohydrolases, a glycerol-dehydrogenase and a glycolate oxidase, further extending its metabolic and adaptive potential for the host. Like in the case of the ICE*Pni*2 (described further below), we hypothesize that the lipid- and fatty-acid-rich environment of the wastewater treatment plant has been selective to fix the acquired lipid metabolism-encoding GI*Pni*1 in the genome of *P. nitroreducens* HBP1.

### 3.4. Defining the Borders of ICEPni1 and ICEPni2

**Figure 2:**
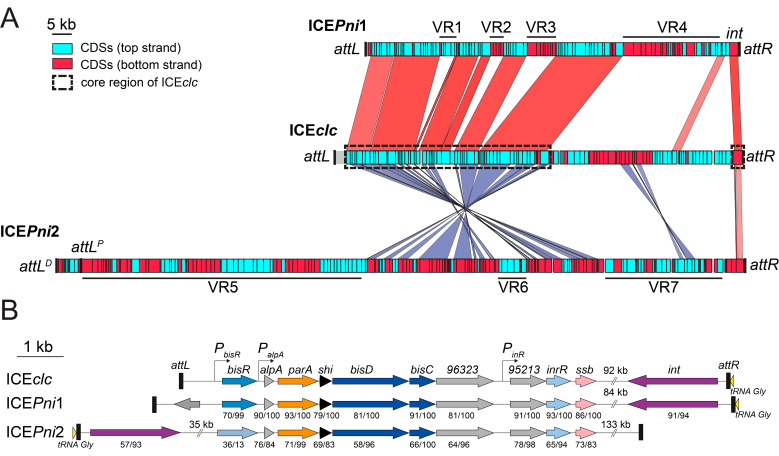
Comparison of ICE*Pni*1 and ICE*Pni*2 from *P. nitroreducens*, and ICE*clc* from *P. knackmussi* B13 (**A**). Linear map of ICE*Pni*1, ICE*Pni*2, and ICE*clc* with indication of the location of predicted ORFs on the top strand (light blue boxes) and bottom strand (red boxes), the integrase-encoding gene (*int*), the attachment sites (*att*, black boxes), and variable regions (VR). The core region of ICE*clc* was framed with dashed black rectangles. The integrase gene of ICE*clc* on the rightmost end of the element was used as a reference point for the alignments. Comparisons were performed using BlastN and are displayed by colored areas linking related regions in the same (red) and inverted (purple) orientation. The intensity of the colored area reflects the percentage of nucleotide identity (minimum 65%) between the sequences. (**B**). Schematic representation (drawn to scale) of the genetic organization of ICE*clc*, ICE*Pni*1 and ICE*Pni*2 regulation loci. Genes are represented by arrowed boxes, and color-coded according to bioinformatic prediction or experimental demonstration of their function: purple, integration/excision; orange, active partition; light to dark blue, transcriptional activators; black, toxic effect; pink, single-stranded DNA protection; light yellow, tRNA; gray, unknown function. Promoters are represented by angled arrows pointing towards the transcription orientation. Numbers under genes (x/y) indicate the percentage of amino acid identity (x) and the coverage (y) of corresponding gene product in ICE*clc*.

Both putative ICE regions in the *P. nitroreducens* HBP-1 genome contained an integrase gene. The ICE*Pni*1 integrase gene (G5B91_21265) shared 91% aa identity over 94% of the protein sequence with that of the ICE*clc* and was oriented adjacent to and in the same orientation as a tRNA operon encompassing three GCC *tRNA^Gly^* and one TTC *tRNA^Glu^* gene (G5B91_21285-G5B91_21270). This strongly resembled the ICE*clc* “right end” structure (Figure 2) [34]. A repeat of the 18-bp 3′-end sequence of the *tRNA^Gly^* (5′-G(A/T)CTCGTTTCCCGCTCCA-3′) was found about 95 kb upstream, suggesting that these repeats form *attR* and *attL* recombination sites (Figure 1, Figure 2A, and Table 4). The size of ICE*Pni*1 in between the two 18-bp repeats was 95,926 bp (chromosome coordinates 4,376,070–4,471,995). The ICE*Pni*1 attachment sites were almost identical to those of ICE*clc*, suggesting that they share a similar integration specificity [34].

The ICE*Pni*2 integrase (G5B91_07495) showed 57% aa identity to IntB13 over 93% of the predicted protein sequence. The gene was located on the minus strand and was preceded by a CCC *tRNA^Gly^* gene (G5B91_07500). Two sequences identical to the *tRNA^Gly^* 18-bp 3′-end (5′-TTCCCTTCGCCCGCTCCA-3′) were found about 170 kb upstream, in two direct copies separated by ca. 6 kb from each other. We designated these as candidate *proximal* and *distal attL* recombination sites (*attL^P^*_ICE*Pni*2_ and *attL^D^*_ICE*Pni*2_ respectively) (Figure 2A and Table 4). As we show below, we only detected excision from the *proximal* recombination site *attL^P^*_ICE*Pni*2_ and identified its recombination from post-transfer integration in the new host. This indicated, therefore, that ICE*Pni*2 would encompass the region 1410,790–1582,233 of the *P. nitroreducens* HBP-1 chromosome, and its size, including both repeats, would amount to 171,444 bp (Figure 2A).

### 3.5. ICEPni1 and ICEPni2 Are Related to ICEclc

Both ICE*Pni*1 and ICE*Pni*2 showed a region with extensive similarity to the core gene region of ICE*clc*, which codes for functions essential to the ICE lifestyle such as integration, excision, DNA processing, mating pore formation, and regulation (Figure 2A, dashed black rectangles) [44]. The analogous core region of ICE*Pni*2 appears to be ‘inverted’ relative to the location of the integrase gene on ICE*clc* and ICE*Pni*1 (Figure 2A). Homologs of the main regulatory genes for ICE*clc* activation were present on ICE*Pni*1 and ICE*Pni*2 (Figure 2B) [45]. This notably included a BisR activator homolog on ICE*Pni*1 with 70% aa identity over 99% of the sequence related to BisR of ICE*clc*, and further, homologs of *alpA*, *parA*, *shi*, *bisD* and *bisC*. The strong gene synteny between regulatory loci suggests that ICE*Pni*1 may be activated in a similar manner as ICE*clc*, which would thus entail a bistable formation of transfer competence in a specific small subpopulation of cells [45,46,47]. Gene homologs on ICE*Pni*2 were more distantly related to the ICE*clc* regulatory module with around 70% nucleotide and 59–76% amino acid sequence identity, but still conserved synteny of *alpA* throughout *bisC*. In contrast, ICE*Pni*2 did not appear to encode a BisR regulator homolog upstream of *alpA*, suggesting that its activation may proceed differently than for ICE*Pni*1 and ICE*clc* (Figure 2B).

Comparison of the ICEs of *P. nitroreducens* with ICE*clc* further showed how conserved stretches are interspersed with unique variable regions (VRs), highlighting the modular pattern of their evolution (e.g., VR-regions indicated in Figure 2A). Some of the variable regions may have been maintained because they conferred selective advantages to *P. nitroreducens*, as will be discussed further below.

### 3.6. ICEPni1 Encodes Mercury, Arsenic and Formaldehyde Detoxification and a Bacteriophage Defense System

Manually curated annotations (Table S1, sheet ICE*Pni*1) suggested that ICE*Pni*1 carries three sets of genes for defense against toxic compounds. The first set (on VR1, G5B91_20860 to G5B91_20875) encodes the archetypal mercury resistance determinants. This includes mercury(II) uptake and reduction by a complex of mercuric transport protein MerT, mercuric transport protein periplasmic component MerP, mercuric reductase MerA under control of Hg(II)-responsive transcriptional regulator MerR. The second set (i.e., VR2, G5B91_20945 to G5B91_20965) codes for arsenate/arsenite resistance, with a classical transcriptional repressor ArsR, which controls an operon of arsenate reductase ArsC and arsenite efflux transporter ArsB. These, together, facilitate reduction of As(V) to As(III) and its efflux, followed by a trivalent organoarsenical oxidase ArsH, which oxidizes methyl-arsenite As(III) and other highly toxic trivalent organoarsenicals (e.g., herbicides MSMA, DSMA or CAMA) to less toxic pentavalent species [48,49]. The group of genes within VR4 (Figure 2A) likely codes for formaldehyde oxidation via glutathione-dependent and –independent pathways, and is represented by homologues of S-(hydroxymethyl) glutathione dehydrogenase FrmA (G5B91_21165) and S-formylglutathione hydrolase FrmB (G5B91_21180) on one hand, and formaldehyde dehydrogenase FdhA (G5B91_21240) on the other. VR4 also carried a mosaic of genes and remnants thereof belonging to different functional categories, e.g., carbon metabolism (notably, dye decolorizing peroxidase, nitrilase/amidase and oxidoreductase), DNA recombination and repair (replication-associated recombination protein A, excinuclease components UvrA and UvrB, two transposases), and a variety of transcriptional regulators (Table S1, sheet ICE*Pni*1). In accordance with these predictions, *P. nitroreducens* was able to grow in the presence of up to 8 µM of mercury chloride and 10 mM sodium arsenite, and it transferred the mercury resistance trait via conjugation (see below). Finally, another feature of adaptive significance is encoded on VR3; an operon spanning loci G5B91_21005 to 21040 (Table S1, sheet ICE*Pni*1), which encodes proteins resembling the recently described cyclic oligonucleotide-based anti-phage signaling system (CBASS) [50]. Taken together, the ICE*Pni*1 cargo is densely packed with functions of detoxification and defense, which may have been advantageous for the survival of *P. nitroreducens* in the polluted (wastewater) environment from whence it was isolated.

### 3.7. ICEPni2 Encodes Metabolism of Aromatic Compounds and Fatty Acids

ICE*Pni*2 contains genes for two complete central pathways for aromatic compound metabolism: a catechol *meta*-cleavage pathway (G5B91_07410 to G5B91_07475, 14 genes) and a protocatechuate *ortho*-cleavage pathway (G5B91_06945 to G5B91_07000, 12 genes) (Table S1, sheet ICE*Pni*2). In addition, genes for so-called peripheral pathways encode the enzymes to channel mono- and di-aromatic substrates into the central pathways. Among these, the previously characterized *hbpRCAD* genes (G5B91_07355 to G5B91_07370) are responsible for transforming 2-hydroxy- or 2,2’-dihydroxy-biphenyl to 2-hydroxy-2,4-pentadienoic acid and either benzoate, or salicylate, respectively [6,14]. 2-hydroxy-2,4-pentadienoic acid enters the *meta*-pathway directly, whereas benzoate is first converted to catechol by action of a benzoate 1,2-dioxygenase and 1,6-dihydroxycyclohexa-2,4-diene-1-carboxylate dehydrogenase encoded by the host genome loci G5B91_12935-G5B91_12945 (not on ICE*Pni*1). Salicylate may be decarboxylated to catechol by the ICE*Pni*2-encoded salicylate hydroxylase (G5B91_07395). Further peripheral pathways encoded on ICE*Pni*2 consist of a ferulate-CoA synthase FerA G5B91_06740, vanillin synthase/trans-feruloyl-CoA hydratase FerB G5B91_06745, vanillin dehydrogenase G5B91_06735, vanillate O-demethylase VanAB G5B91_06885-G5B91_06890 and 4-hydroxybenzoate 3-monooxygenase PobA G5B91_06935. These may catalyze the channeling of ferulate, vanillin, vanillate and 4-hydroxybenzoate into the protocatechuate pathway (Table S1, sheet ICE*Pni*2). Finally, genes for a two-component aromatic hydroxylase G5B91_06770 and G5B91_06795, an FMN-dependent NADH-azoreductase (G5B91_06765), and for carbazole 1,9a-dioxygenase components G5B91_07470 and G5B91_07475, may imply the potential of *P. nitroreducens* to metabolize other undefined aromatic substrates. Interestingly, the genes for a major MexAB-OprM efflux system that is crucial to grow on 2HBP are not present on ICE*Pni*2, but elsewhere on the chromosome (G5B91_02490–02510) [51].

A second large group of ICE*Pni*2 catabolic functions is represented by around 30 genes organized in several transcriptional units (Figure 2A, VR5), which may encode transport and metabolism of fatty acids. Notably, all genes are present for the enzymes (some redundant) required for fatty acid beta-oxidation: the long-chain-fatty-acid-CoA ligase FadD (two ORFs), acyl-CoA dehydrogenase FadE (four ORFs), enoyl-CoA hydratase/isomerases FadB (two ORFs) and acetyl-CoA C-acyltransferases/thiolases FadA (two ORFs). Furthermore, ICE*Pni*2 also encodes a 3-oxoacyl-ACP reductase FabG and enoyl-[acyl-carrier-protein] reductase FabI, which are involved in fatty acid synthesis and acyl-carrier-protein metabolism (Table S1, sheet ICE*Pni*2). These genes were intermingled with ORFs encoding NAD- and FAD-dependent oxidoreductases, electron transfer proteins, hydrolases, transporters, transcriptional regulators, chemotactic and DUF-proteins (Table S1, sheet ICE*Pni*2), which often co-occur with genes for fatty acid metabolism in other reference genomes, and on GI*Pni*1 (data not shown). The presence of these functions on ICE*Pni*2 may have provided a selective advantage in the fatty acid-rich sewage environment (as we discussed above for GI*Pni*1) and, possibly, also to connect the acetyl-CoA-producing aromatic catabolic pathways to the central cell metabolism. Finally, close to the left end proximity of ICE*Pni*2, we identified homologues of the *imuA-imuB-imuC (dnaE2)* gene cassette (G5B91_06695-G5B91_06705), which may encode an error-prone translesion DNA synthesis polymerase complex [52]. ImuABC polymerases are regulated by the SOS-response and facilitate replication bypass of damaged DNA, concomitantly enhancing spontaneous mutagenesis [53,54,55]. These are properties that might be advantageous for both resilience and for accelerated adaptive evolution to the strongly selective environments in which *P. nitroreducens* has prevailed. The variable gene content of ICE*Pni*2 seemed “patchy”, i.e., indicative of their recent acquisition via multiple recombination events and from different donor genomes. The number of recombination-promoting sequences on ICE*Pni*2 is limited to only one intact (*istAB*) and two mutated transposase genes (Table S1, sheet ICE*Pni*2), suggesting that other recombination mechanisms may have prevailed in the acquisition of the variable gene content.

### 3.8. ICEPni1 Can Excise and Transfer to *P. putida*, and Integrates into One of Four tRNA^Gly^ Gene Targets

In order to test whether the identified ICEs were functional, we first examined their possible excision from the chromosome as a circular element carrying an *attP* attachment site [56]. A weak but reproducible signal for an amplified DNA fragment covering both extremities of ICE*Pni*1 and containing its predicted *attP_ICEPni_*_1_ site was obtained by PCR on DNA extracted from *P. nitroreducens* grown in MM with 20 mM succinate with or without 5 mM sodium arsenite or 4 µM mercury chloride or in MM with 5 mM 2HBP. The PCR amplicon was visible in DNA from three independent cultures that were sampled in exponential, early and late stationary growth phase (data not shown). This showed that ICE*Pni*1 excision did occur, albeit at a low frequency. Subsequent amplicon sequencing confirmed that the *attP* site was formed by recombination at the inferred 18-bp repeats (Table 2 and Table 4).

To test the potential of ICE*Pni*1 to transfer, we filter-mated *P. nitroreducens* with *P. putida* UWCGC as a recipient while selecting for mercury resistance (at 4 µM). *P. putida* UWCGC transconjugants resistant to mercury appeared at a frequency of 1.16 (±0.37) × 10^−6^ per donor CFU. The transconjugants were further Gm resistant and mCherry fluorescent, indicating they were genuine UWCGC derivatives. Among fifty colonies tested with PCR, nine (18%) amplified a fragment of the *merA* gene specific for ICE*Pni*1 (primers pPazICE1_merA_fw/pPazICE1_merA_rev, Table 1), which confirmed transfer of ICE*Pni*1 (with *merA*). The large proportion of mercury-resistant colonies without amplifiable *merA* of ICE*Pni*1 suggested transfer of (an)other DNA element(s) from *P. nitroreducens* HBP-1 conferring mercury resistance. Our attempt to specifically amplify the *merA* allele carried by plasmid pPniHBP1_1 yielded no product, thus ruling out the transfer of this plasmid. Possibly, the second plasmid pPniHBP1_2 (Figure 1C) was transferred, which carries genes for ZntA-like P-type ATPase and a CzcCBA-like tripartite cation efflux system that might confer mercury resistance, but this was not tested.

In seven *P. putida* UWCGC transconjugants carrying *merA* of ICE*Pni*1, we further analyzed the potential integration events. ICE*Pni*1 had integrated into any of the four GCC *tRNA^Gly^* genes on *P. putida* (Table 4, locus tags PP_t21, _t23, _t24, and _t59), exactly as was previously demonstrated for its sibling ICE*clc* [34]. Sequencing of the recombination site boundaries showed that *attL* retained the 18 bp sequence of the ICE*Pni*1 *attP*-site (G**A**CTCGTTTCCCGCTCCA, Table 4). This sequence has one mismatch with respect to *attB* in *P. putida* (G**T**CTCGTTTCCCGCTCCA), and to *attR* of ICE*Pni*1 in *P. nitroreducens* HBP-1 (Table 4). All four sequenced *attL* sites of transconjugants contained this same nucleotide difference. This indicates that both excision and integration catalyzed by the ICE*Pni*1 integrase can tolerate (some) mismatches in the recombination sites, thus likely allowing ICE establishment in broader range of hosts.

### 3.9. ICEPni2 Is also a Functional ICE

Excision of ICE*Pni*2 was confirmed by PCR amplification of the *attP* junction in DNA extracted from cultures of *P. nitroreducens*. Interestingly, we could amplify a DNA fragment predicted to contain the junction between the proximal 18-bp repeat and *attL* (see above), but not the distal repeat. The junction was confirmed by sequencing (Table 2 and Table 4). Clear PCR amplification was detected on a gel only in two out of nineteen DNA samples, suggesting a generally low incidence of excision (data not shown).

Selection of *P. putida* UWCGC transconjugants from matings with *P. nitroreducens* HBP-1 on minimal medium agar with 5 mM 2-HBP and Gm yielded no colonies, possibly because of the toxicity of 2-HBP [51]. In contrast, selection on 2.5 mM salicylate and Gm yielded eight colonies among 8.6 × 10^8^ donor cells, which would be equivalent to a transfer rate of 10^−8^ per donor CFU. From the DNA of all eight colonies, we could amplify a fragment of the *hbpA* gene, which is specific to ICE*Pni*2. All colonies were also mCherry fluorescent, confirming that they are *P. putida* recipients. This also confirmed that ICE*Pni*2 is transferable from *P. nitroreducens*. All recovered transconjugants had integrated ICE*Pni*2 at the 3′-end of the CCC *tRNA^Gly^* gene (locus_tag = PP_t64), suggesting a high specificity for this integration site. Interestingly, the presumed 18-bp *attP* site of ICE*Pni*2 and the targeted *attB* site in *P. putida* had three mismatches, which resulted in different combinations of *attL*/*attR* sites flanking the integrated element in *P. putida* (Table 4). Recombination involving two non-identical sequences is known to result in the formation of a heteroduplex that is resolved in a variety of combinations of *att* sites in the integrated form of ICE*Pni*2, which is not uncommon for tyrosine-type recombinases [57]. The low transfer rate may thus stem from a lower incidence of excision, absence of an efficient efflux detoxification system to allow productive growth on 2-HBP and salicylate [51], and from mismatch between the ICE*Pni*2 *attP* site and the *P. putida attB* site that limits efficient recombination.

To verify the functionality of ICE*Pni*2-encoded aromatic catabolism genes, we tested the ability of *P. nitroreducens* HBP-1 (ICE donor), *P. putida* UWCGC (recipient) and two *P. putida* UWCGC (ICE*Pni*2) transconjugants to grow aerobically in minimal medium supplemented with 2.5 mM 2-HBP, salicylate, 4-hydroxybenzoate and vanillate as the sole carbon source. As expected, *P. nitroreducens* HBP-1 and *P. putida* UWCGC ICE*Pni*2-transconjugants, but not *P. putida* UWCGC, were able to grow on 2-HBP and salicylate. This supported our hypothesis that ICE*Pni*2 indeed contains all necessary genetic information for these two metabolic pathways. The above-mentioned inability to select for ICE*Pni*2 transconjugants using 2-HBP most likely reflected the need for the *P. putida* recipient cells to integrate ICE*Pni*2 catabolism into its own metabolic network before being able to grow on 2-HBP. In contrast to *P. nitroreducens* HBP-1, *P. putida* UWCGC ICE*Pni*2-transconjugants did not grow on 4-hydroxybenzoate. This would indicate that the ICE*Pni*2 transferred protocatechuate pathway may not have been functionally expressed in *P. putida*.

## 4. Concluding Remarks

The gapless sequence of the *P. nitroreducens* HBP-1 genome gives a clear view of the numerous gene acquisitions and adaptations that may have provided HBP-1 with selective advantages to cope with the polluted environment from whence it was isolated.

The exploration of HBP-1′s genomic plasticity indicated a variety of genetic elements that were most likely acquired by horizontal gene transfer, some of which (both ICEs) were capable of conjugative transfer from HBP-1 as donor. In contrast to previous reports [15], HBP-1 carries not only a single large plasmid but a second plasmid with a variety of potential adaptive functions (Figure 1B,C and Table S1). It will be interesting to learn more about the capability and mode of dissemination of these plasmids, and the importance of their adaptive gene functions to the host.

The *P. nitroreducens* HBP-1 genome is scattered with putative prophages, genomic islands and ICEs, most of which with unknown properties, origins and functionalities. A 126 kb long genomic island (GI*Pni*1) was identified with unclear motility properties, as well as several putative prophages, or derivative elements whose mobility is compromised or dependent on helper element(s) (Figure 1 and Table 3) [36,58]. The two discovered ICEs, ICE*Pni*1 and ICE*Pni*2, code for many obvious adaptive functions, and confer *P. nitroreducens* with the capability to resist to heavy metals and to metabolize aromatic compounds, respectively (Figure 2A). Interestingly, both elements are related in their core structure to the prototypical element ICE*clc* (Figure 2), indicating the importance and wide distribution of this type of element among Gammaproteobacteria. In contrast to ICE*clc*, ICE*Pni*1 and ICE*Pni*2 transfer at much lower frequencies (10^−6^–10^−8^ per donor, compared to 10^−2^ for ICE*clc*), but we demonstrated their excision, transfer and integration in the chromosome of *P. putida*. They are therefore fully functional ICEs. Given the bistable development of transfer competence imposed by ICE*clc* on its host, it will be interesting to see if ICE*Pni*1 and ICE*Pni*2 act similarly. Such a mechanism is pivotal for the lifecycle of ICE*clc* [46,47,59], and differences and/or similarities in the regulation of ICE*Pni*1 and ICE*Pni*2 would strengthen our understanding of bistable behavior [45].

## Figures and Tables

**Table 1:** Primers used in this study.

Name	Sequence (5′-3′)	Reference
pPaz2002_fw	CACAGGCGCTTTTGCTTGC	This work
pPaz2003_rev	CAGATGGTTGATGTAGCCGATAG	This work
pPazICE1_left_fw	GACTCGGGGCGATCCATTGAC	This work
pPazICE1_left_rev	CGCGACCCGTGCTCAAAC	This work
pPazICE1_merA_fw	ATCATGGCCGAGGCGATCAC	This work
pPazICE1_merA_rev	GCCAGGATGCCTTCGTACTTGG	This work
pPazICE2_right2_fw	GAACCGTGAGGTCTGAGAGCATG	This work
pPazICE2_right_rev	CCACTTCATCGACGTGGAACACA	This work
pPazICE2_LE_DisFw	CCTGACGCTGCCATCTGCCT	This work
pPazICE2_LE_DisRe	GAATGGCTGCAATCAGGAACGAC	This work
pICE2_LeftProxOut	CCGCCAGCACCCACAGAC	This work
pICE2_LeftProxIn	CATCTTGGAGTAAGAGCTGCGC	This work
pPazICE2_hbpA_fw	GGGTGCTGGTCCGGCTGG	This work
pPazICE2_hbpA_rev	CGGCGTTTTGCGCAACTAGC	This work
Pgly1fw	GCCCAAGCGTCGTGATGAATG	[34]
Pgly1rev	ACGTGAGCGGTGTTGATGGTGAT	[34]
Pgly2rev	TTGAAGCGGATCGGTGGGTAAT	[34]
Pgly2fw	AATGTCATGCTGGGCTTCCTCAA	[34]
Pgly3fw	CAGTAATGCCAGCAGCGTGTCC	[34]
Pgly3rev	GTGCCGGAGAAACTGGAGCG	[34]
Pgly4fw	ATCGTGAGGTTCATGTTCTGGTGC	[34]
Pgly4rev	GCACCGCATAGACGCCACAGTA	[34]
Pgly5fw	TCACGCCGAACGTGGTAAAGC	[34]
Pgly5rev	GACCTCCGTGGAAGGCTGTAAATCT	[34]
Pgly6fw_SV 070933	TCGCTAGAATGGCACCCATCAC	[34]
Pgly6rev 060353	CGCCGCGTTGTGGTGTTTG	[34]
Pgly6fw 060354	TCCTGCTCATTCCGTGCTTCATT	This work
Pgly6rev2 140309	CGACTGAAACCTGTAGATC	This work

**Table 2:** List of primers used to amplify integrative and conjugative element (ICE) recombination sites.

Recombination/Integration Site	Primer Pair	Amplicon Size (bp)
**ICE*Pni*1 in *P. nitroreducens***		
*attR*_ICE*Pni*1_	pPaz2002_fw/pPaz2003_rev	1138
*attL*_ICE*Pni*1_	pPazICE1_left_fw/pPazICE1_left_rev	984
*attP*_ICE*Pni*1_	pPaz2002_fw/pPazICE1_left_rev	982
**ICE*Pni*2**		
*attR*_ICE*Pni*2_	pPazICE2_right2_fw/pPazICE2_right_rev	933
*attL^P^*_ICE*Pni*2_	pICE2_LeftProxOut/pICE2_LeftProxIn	834
*attL^D^*_ICE*Pni*2_	pPazICE2_LE_DisFw/pPazICE2_LE_DisRe	934
*attP^P^*_ICE*Pni*2_	pPazICE2_right2_fw/pICE2_LeftProxOut	717
*attP^D^*_ICE*Pni*2_	pPazICE2_right2_fw/pPazICE2_LE_DisFw	934
***attB* Integration Sites in *P. putida* UWC1 ^a^, *tRNA^Gly^***		
*tRNA^Gly^*-1 PP_RS02330	Pgly1fw/Pgly1fw	985
*tRNA^Gly^*-2 PP_RS07265	Pgly2fw/Pgly2rev	401
*tRNA^Gly^*-3 PP_RS09655	Pgly3fw/Pgly3rev	185
*tRNA^Gly^*-4 PP_RS09665	Pgly4fw/Pgly4rev	225
*tRNA^Gly^*-5 PP_RS09670	Pgly5fw/Pgly5rev	135
*tRNA^Gly^*-6 PP_RS21295	Pgly6fw/Pgly6rev	803
**ICE*Pni*1 Junctions in *P. putida***		
ICE*Pni*1-*tRNA^Gly^*-3 (*attR*)	Pgly3fw/pPaz2002_fw	553
ICE*Pni*1-*tRNA^Gly^*-4 (*attR*)	Same as above	800
ICE*Pni*1-*tRNA^Gly^*-5 (*attR*)	Same as above	962
ICE*Pni*1-*tRNA^Gly^*-6 (*attR*)	Pgly6fw_SV/pPaz2002_fw	482
ICE*Pni*1-*tRNA^Gly^*-3 (*attL*)	pPaz2002_rev/Pgly3rev	1039
ICE*Pni*1-*tRNA^Gly^*-4 (*attL*)	pPaz2002_rev/Pgly3rev	792
ICE*Pni*1-*tRNA^Gly^*-5 (*attL*)	pPaz2002_rev/Pgly3rev	637
ICE*Pni*1-*tRNA^Gly^*-6 (*attL*)	pPaz2002_rev/Pgly6rev2	607
**ICE*Pni*2 Junctions in *P. putida***		
ICE*Pni*2-*tRNA^Gly^*-2 (*attR*)	pPazICE2_right2_fw/Pgly2fw	536
ICE*Pni*2-*tRNA^Gly^*-2 (*attL*)	pICE2_LeftProxOut/Pgly2rev	579

^a^ Locus tag are based on the *P. putida* KT2440 reference genome accession NC_002947.4.

**Table 3:** PHASTER predictions of prophage locations in *P. nitroreducens* HBP-1.

Replicon	Phage	Start-Stop (bp)	Size (kb)	Prediction ^a^	Related Phage-Accession Number
**Chromosome**	P1	211,373–255,489	44.1	intact	*Stenotrophomas* phage S1-NC_011589(5)
	P2	654,715–672,064	17.3	intact	*Enterobacter* phage Arya-NC_031048(6)
	P3	1,611,889–1,643,994	32.1	incomplete	*Pseudomonas* phage FHA0480-NC_041851(8)
	P4	2,297,162–2,315,460	18.3	incomplete	*Pseudomonas* phage YMC11/02/R656-NC_028657(7)
	P5	2,319,087–2,355,574	36.5	questionable	*Pseudomonas* phage PAJU2-NC_011373(14)
	P6	2,525,149–2,570,649	45.5	intact	*Pseudomonas* phage MD8-NC_031091(5)
	P7	2,562,995–2,608,469	45.5	intact	*Pseudomonas* phage phiCTX-NC_003278(29)
	P8	2,935,034–2,974,747	39.7	intact	*Pseudomonas* phage JBD25-NC_027992(34)
	P9	3,666,393–3,701,365	35.0	intact	*Salmonella* phage SEN34-NC_028699(20)
	P10	3,714,527–3,770,149	55.6	intact	*Salmonella* phage SEN34-NC_028699(19)
	P11	5,332,674–5,379,949	47.3	intact	*Pseudomonas* phage YMC11/02/R656-NC_028657(9)
**pPniHBP1_1**	P12	144,536–158,207	13.7	incomplete	*Acinetobacter* phage vB_AbaM_ME3-NC_041884(3)
	P13	323,653–329,028	5.4	incomplete	*Pseudomona*s phage MD8-NC_031091(2)
	P14	385,628–394,444	8.8	incomplete	Halovirus HCTV5-NC_021327(2)
**pPniHBP1_2**	P15	51,522–65,829	14.3	incomplete	*Ralstonia* phage p12J-NC_005131(2)

^a^ Incomplete, questionable and intact refer to PHASTER total score [31].

**Table 4:** Sequenced attachment sites of ICE*Pni*1 and ICE*Pni*2 in *P. nitroreducens* HBP-1 and in *P. putida* UWCGC.

Attachment Site	Sequence (5′ to 3′)	Host
**ICE*Pni*1**		
*attL*	G**A**CTCGTTTCCCGCTCCA	*P. nitroreducens*
*attR*	G**T**CTCGTTTCCCGCTCCA	*P. nitroreducens*
*attP*	G**A**CTCGTTTCCCGCTCCA	*P. nitroreducens*
*attB*	G**T**CTCGTTTCCCGCTCCA	*P. nitroreducens*
*attB*	G**T**CTCGTTTCCCGCTCCA	*P. putida*
*attL*	G**A**CTCGTTTCCCGCTCCA	*P. putida*
*attR*	G**T**CTCGTTTCCCGCTCCA	*P. putida*
**ICE*Pni*2**		
*attL*	TTCCCT**TCG**CCCGCTCCA	*P. nitroreducens*
*attR*	TTCCCT**TCG**CCCGCTCCA	*P. nitroreducens*
*attP*	TTCCCT**TCG**CCCGCTCCA	*P. nitroreducens*
*attB*	TTCCCT**TCG**CCCGCTCCA	*P. nitroreducens*
*attB*	TTCCCT**CTA**CCCGCTCCA	*P. putida*
*attL1*	TTCCCT**CTA**CCCGCTCCA	*P. putida*
*attL2*	TTCCCT**TCG**CCCGCTCCA	*P. putida*
*attR1*	TTCCCT**CTA**CCCGCTCCA	*P. putida*
*attR2*	TTCCCT**TCG**CCCGCTCCA	*P. putida*

## Supplementary Materials

The following are available online at https://www.mdpi.com/2073-4425/11/8/930/s1, Table S1: *P. nitroreducens* HBP-1 genome annotation, Figure S1: Read coverage plot of the *P. nitroreducens* HBP-1 genome. Graphical representation of the moving-average coverage per 100-bp window obtained from Illumina sequencing of *P. nitroreducens* HBP-1 replicons: chromosome (in blue), pPniHBP1_1 (in green) and pPniHBP1_2 (in red) using 1 colony (A. PNI1) or 5 colonies (B. PNI5) to inoculate an overnight culture. The mean and standard deviation of coverage are indicated for each replicon.
