# Supplementary figures and images for: Insights into Mobile Genetic Elements of the Biocide-Degrading Bacterium Pseudomonas nitroreducens HBP-1
Source: Genes (Basel). 2020 Aug 12;11(8):930. doi: 10.3390/genes11080930 (PMC7466150; doi:10.3390/genes11080930)

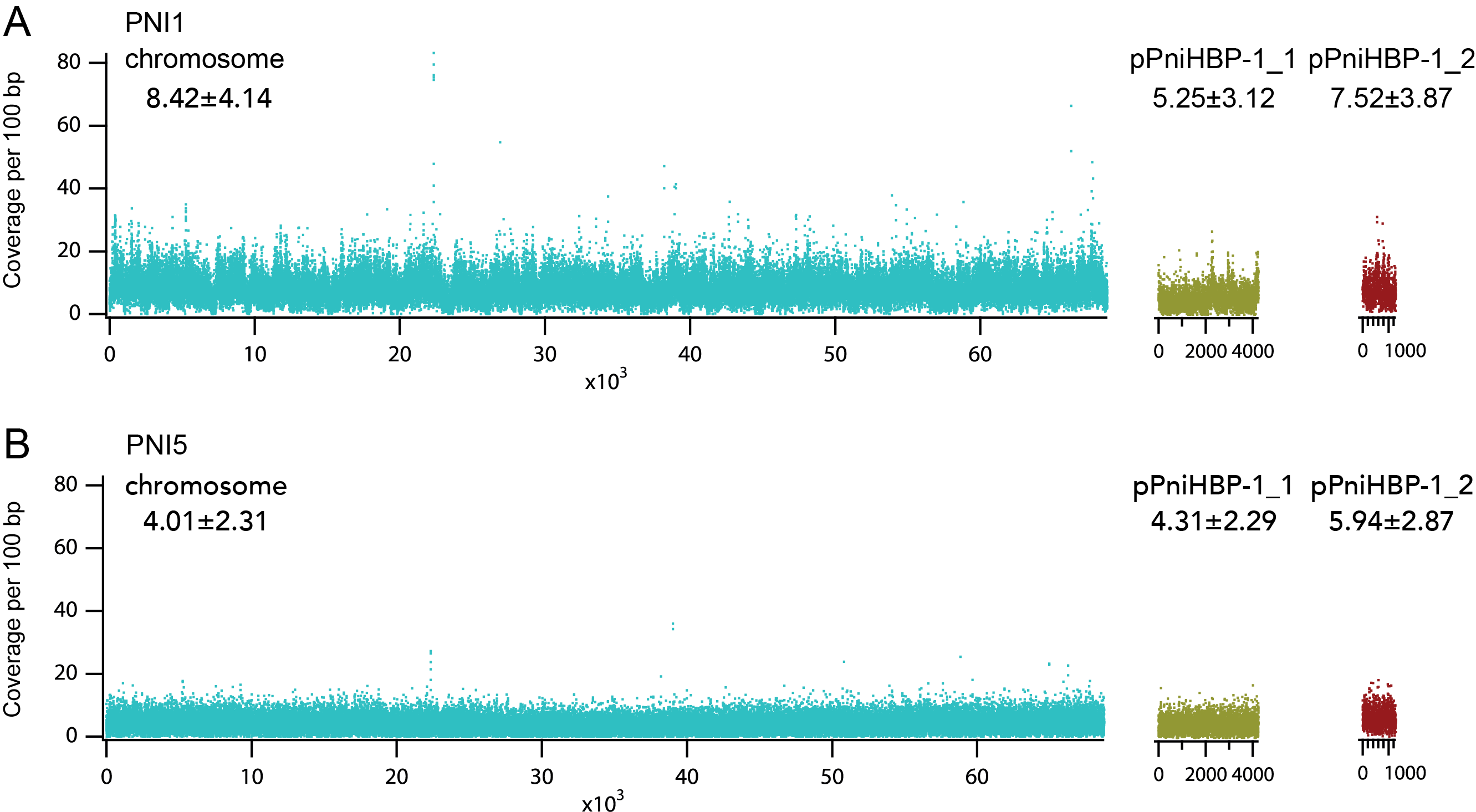

Supplement: Supplementary file 1 [file genes-11-00930-s001.zip › supp/FigS1.png]
